# ID 211 - Caracterização Preliminar da Contribuição dos Dados do SUS para Modelos de Difusão

**DOI:** 10.5327/2237-9622.2025.v34s1.211

**Published:** 2025-11-25

**Authors:** Ariane Gonçalves Silva de Araujo, Pietro Lucas Agner Garmatter, Layssa Andrade Oliveira, Fernanda Stumpf Tonin, Rosa Camila Lucchetta, Astrid Wiens

**Keywords:** difusão de inovações, avaliação de tecnologias em saúde, mineração de dados

## Abstract

**Introdução:**

Análises de registros administrativos de dispensação de medicamentos podem ser uma importante fonte de informações para aprimorar métodos de estimativas de taxa de difusão de novas tecnologias, reduzindo as incertezas em análises de impacto orçamentário, ao fornecer dados mais fidedignos ao que ocorre na prática clínica após a incorporação de um medicamento. Neste resumo, fornecemos uma caracterização preliminar da contribuição dos registros de dispensação de medicamentos no Sistema Único de Saúde no Brasil (SUS) no contexto de desenvolvimento de modelo de predição da taxa de difusão.

**Método:**

Foram selecionados todos os medicamentos incorporados no SUS, normatizados nos Protocolos Clínicos e Diretrizes Terapêuticas (PCDT) do Ministério da Saúde (MS) e pertencentes ao grupo 1A do Componente Especializado da Assistência Farmacêutica (Ceaf), conforme a Relação Nacional de Medicamentos Essenciais (Rename) 2022. Os registros de dispensação mensal entre janeiro de 2008 e junho de 2022 foram provenientes do Departamento de Informação e Informática do SUS (DataSUS), conforme o processo da Sala Aberta de Inteligência em Saúde (Sabeis).

**Resultados:**

Foram identificados 63 PCDT publicados pelo MS, abrangendo 83 medicamentos financiados como Ceaf 1A. Destes, 59% dos PCDT apresentaram apenas um medicamento Ceaf 1A incorporado. Dos 83 medicamentos incluídos para análise, 33% estavam normatizados em dois ou mais PCDT, enquanto 67% tinham um único PCDT correspondente. Os PCDT com maior número de medicamentos normatizados do grupo 1A do Ceaf eram relacionados à esclerose múltipla e às doenças reumáticas, como artrite reumatoide, artrite psoríaca, espondilite ancilosante e artrite idiopática juvenil. Entre os medicamentos, o metotrexato foi o mais frequente, normatizado em 9 PCDT, seguido pelo adalimumabe e pela imunoglobulina humana de 5 g, presentes em 8 PCDT. Até junho de 2024, foram analisados 36 PCDT: 1 com 7 medicamentos normatizados e 35 com apenas 1 medicamento normatizado. Em relação aos modelos de difusão, foi possível identificar um modelo explicativo da difusão real para 55,6% dos PCDT. Contudo, para 16,7% deles, o modelo explicou parcialmente a difusão ocorrida, indicando a necessidade de mais investigação. Além disso, em 27,8% dos PCDT, os dados de utilização foram insuficientes para o desenvolvimento da modelagem de séries temporais.

**Conclusão:**

Até o momento, a escassez de dados de dispensação foi o principal fator limitante na identificação de modelos de difusão. No entanto, a identificação de modelos explicativos da difusão real em mais da metade dos PCDT analisados já aponta para uma importante contribuição desses registros na busca por ferramentas mais precisas de predição da difusão, especialmente no contexto de medicamentos sem concorrente farmacológico no momento da incorporação. Análises futuras de PCDT com maior número de medicamentos normatizados, apesar de mais complexas, podem trazer mais contribuições por fornecerem mais variáveis explicativas da difusão real desses medicamentos.

